# Functional characterization of cucumber (*Cucumis sativus* L.) Clade V *MLO* genes

**DOI:** 10.1186/s12870-017-1029-z

**Published:** 2017-04-21

**Authors:** Jeroen A. Berg, Michela Appiano, Gerard Bijsterbosch, Richard G. F. Visser, Henk J. Schouten, Yuling Bai

**Affiliations:** Plant Breeding, Wageningen University & Research, Droevendaalsesteeg 1, 6708 PB Wageningen, The Netherlands

**Keywords:** Cucumber (*Cucumis sativus* L.), Powdery mildew, *MLO*, Susceptibility genes, Gene expression

## Abstract

**Background:**

Powdery mildew (PM) causing fungi are well-known pathogens, infecting over 10.000 plant species, including the economically important crop cucumber (*Cucumis sativus* L.). Loss-of-function mutations in clade V *MLO* genes have previously been shown to lead to recessively inherited broad-spectrum resistance to PM in several species. In cucumber, one clade V *MLO* homolog (*CsaMLO8*) was previously identified as being a susceptibility factor to PM. Two other closely related homologs (*CsaMLO1* and *CsaMLO11*) were found, but their function was not yet unravelled.

**Methods:**

*CsaMLO1* and *CsaMLO11* were cloned from cucumber and overexpressed in a tomato *mlo* mutant. The transcript abundances of all three *CsaMLO* genes in different cucumber tissues were quantified using qRT-PCR and RNA-seq, with and without inoculation with the cucumber PM fungus *Podosphaera xanthii*. Allelic variation of *CsaMLO1* and *CsaMLO11* was screened in silico in sequenced cucumber germplasm.

**Results:**

Heterologous overexpression of all three *CsaMLO* genes in the tomato *mlo* mutant restored susceptibility to PM caused by *Oidium neolycopersici*, albeit to a different extent: whereas overexpression of *CsaMLO1* or *CsaMLO8* completely restored susceptibility, overexpression of *CsaMLO11* was only partially able to restore PM susceptibility. Furthermore, it was observed by qRT-PCR and RNA-seq that *CsaMLO8* was significantly higher expressed in non-inoculated cucumber compared to the other two *MLO* genes. However, inoculation with *P. xanthii* led to upregulation of *CsaMLO1*, but not to upregulation of *CsaMLO8* or *CsaMLO11*.

**Conclusions:**

Both *CsaMLO1* and *CsaMLO11* are functional susceptibility genes, although we conclude that based on the transcript abundance *CsaMLO8* is probably the major clade V *MLO* gene in cucumber regarding providing susceptibility to PM. Potential loss-of-function mutations in *CsaMLO1* and *CsaMLO11* have not been identified. The generation and analysis of such mutants are interesting subjects for further investigation.

**Electronic supplementary material:**

The online version of this article (doi:10.1186/s12870-017-1029-z) contains supplementary material, which is available to authorized users.

## Background

Powdery mildew (PM), caused by ascomycete fungi of the order *Erysiphales*, is one of the most well-known plant diseases [1]. PM fungi are able to cause disease on leaves, stems, flowers and fruits of nearly 10.000 different angiosperm plant species, including various economically important plants, such as cucumber (*Cucumis sativus* L.). PM in cucumber can be caused by two different species, *Golovinomyces orontii* and *Podosphaera xanthii*. In greenhouses and open field cultivation in warm regions, *P. xanthii* appears to be the most occurring agent of PM in cucurbits, whereas *G. orontii* is the major PM species on cucurbits grown in the open field in colder regions [2].

According to the gene-for-gene concept, plants have dominantly inherited resistance genes (*R-*genes), encoding R proteins which recognize the products of corresponding avirulence genes (*Avr*-genes) of pathogens, triggering defence responses [3, 4]. Even though this can give a strong, complete resistance, the pathogen can mutate, lose or silence the recognized *Avr* gene to break the resistance, leading to a new virulent race of the pathogen, often within a few years after the commercial introduction of a new *R* gene. The cloning of *R* genes in several plant species has led to the finding that they typically encode receptor proteins of various classes with leucine-rich repeat (LRR) domains [3]. An exception to *R*-gene mediated resistance was discovered in X-ray irradiated summer barley populations in the 1940’s [5]. A recessively inherited monogenic resistance was observed, which was active against all known isolates of barley PM (caused by *Blumeria graminis* f. sp. *hordei*). Later, other alleles at the same genetic locus were obtained in various barley genotypes, including a naturally occurring mutant allele that was found in resistant Ethiopian barley landraces [6]. The durability of this so-called *mlo* (*Mildew Locus O*) resistance is exemplified by the fact that cultivars with this type of resistance have been extensively cultivated since the 1980s without new races of the pathogen breaking the resistance [7]. By positional cloning, the causal gene for *mlo* based resistance was isolated [8]. It was found to encode a plasma membrane-anchored protein with seven transmembrane helices, reminiscent of animal G-protein-coupled receptors [9].

After the barley *MLO* gene was cloned, it was found that mutations in homologs of this gene in other plant species can also lead to recessively inherited resistance to different PM causing fungi. In the model species *Arabidopsis thaliana* T-DNA insertion mutations in three *MLO* homologs contribute to PM resistance, although a mutation in one of the three genes (*AtMLO2*) has a larger effect compared to mutations in the other two genes (*AtMLO6* and *AtMLO12*). The effect of loss of function of *AtMLO6* or *AtMLO12* is only additive in *Atmlo2* background, but not detectable when *AtMLO2* is intact [10]. In several crop species, i.e. tomato [11], pea [12], cucumber [13] and tobacco [14], recessively inherited PM resistance with similar characteristics compared to barley *mlo* resistance was indeed found to be caused by naturally occurring mutations in *MLO* homologs. In other species, e.g. pepper, wheat, apple and grapevine, knockdown of *MLO* homologs by virus induced gene silencing (VIGS) or RNA interference (RNAi) led to PM resistance [15–18]. This indicates that *mlo*-based resistance is very common in plants, rather than a particular oddity occurring in barley. It has been shown that *mlo-*based resistance depends on the formation of cell wall depositions (papillae) by the plant cell directly beneath PM penetration attempts [19]. The molecular basis of *mlo*-based resistance is however yet poorly understood, although it has been shown that it depends on the function of several molecular components, such as the BAX-inhibitor protein (BI-1) which plays a role in control of programmed cell death [20]; on an intact actin cytoskeleton [21] and on the t-SNARE proteins PEN1 (*Arabidopsis*) and ROR2 (barley) involved in targeted exocytosis [22].

Since the year 2000, when the first plant genome sequence was published, i.e. that of the model plant *Arabidopsis thaliana* [23], an increasing number of plant genomes has been sequenced. In all available sequenced plant genomes *MLO* homologs occur as medium sized gene families with seven to thirty-nine *MLO* genes per plant species [24]. In phylogenetic analyses of the *MLO* gene family, it has been found that *MLO* genes can be divided into seven phylogenetic clades, although not all plant species harbour representatives of all clades [25]. The mosses, representing the most basal lineages of land plants, have *MLO* homologs only one of the clades, i.e. clade I. In other lineages of plants, especially in angiosperm species, the *MLO* gene family has diversified. However, several plant species apparently lost genes in some of the *MLO* clades during evolution, such as the monocotyledonous family of the Poaceae which has no *MLO* homologs in clades V and VI, or several dicotyledonous species such as *Arabidopsis thaliana* and tomato which have lost clade IV *MLO* homologs, even though a basal angiosperm species, *Amborella trichopoda,* has homologs of clade I to VI [25].

Not all the *MLO* genes found so far have been characterised as being required for susceptibility towards PM fungi. For most *MLO* genes mutant phenotypes have not been described yet, although there are examples of clade I *mlo* mutants with a defect in root formation [26] and of clade III *mlo* mutants with defects in pollen tube perception [27] and pollen hydration [28]. So far, all *MLO* genes involved in PM susceptibility (i.e. susceptibility genes, S-genes) have been found to group either in clade IV (for monocotyledonous species) or clade V (for dicotyledonous species). It has been shown that heterologous overexpression of the barley clade IV *MLO* gene can functionally complement loss of function mutations in clade V *MLO* genes in tomato [29], exemplifying that although there are significant differences in amino acid sequence between clade IV and V MLO proteins, they are functionally conserved.

In cucumber, the genome sequence of which was published in 2009 [30], thirteen *MLO* homologs have previously been identified. Of these thirteen homologs, three genes were found to group phylogenetically in clade V. These three genes, named *CsaMLO1*, *CsaMLO8* and *CsaMLO11*, should therefore be considered as potential PM S-genes in cucumber [31]. In other cucurbit crops, i.e. melon (*Cucumis melo*), watermelon (*Citrullus lanatus*) and pumpkin (*Cucurbita pepo*), similar numbers of clade V *MLO* genes have been identified, although pumpkin has four rather than three clade V *MLO* genes [32]. Phylogenetic analysis reveals that the last common ancestor of these cucurbit crops already had at least three clade V *MLO* genes.

Of the three cucumber clade V *MLO* genes, *CsaMLO8* has previously been proven to be a susceptibility gene for PM caused by *P. xanthii*. From cucumber genotypes with recessively inherited PM resistance a natural *Csamlo8* mutant allele was cloned [13, 33]. While heterologous overexpression of the wild-type *CsaMLO8* was able to functionally complement *mlo* loss-of-function mutants in both tomato [13] and *Arabidopsis* [33], the mutant allele failed to restore susceptibility. The mutation in *Csamlo8* has been caused by the integration of a retrotransposable element in the coding sequence of the gene [13, 34]. This mutant allele was found to occur frequently in cultivated cucumber germplasm [13], and additionally two other *Csamlo8* loss-of-function mutations have been found in resistant genotypes due to either a frameshift indel leading to an early stop codon, or a SNP in an intron-exon junction causing aberrant splicing of the pre-mRNA [33].

In a review of co-localization of cucumber *MLO* genes with QTLs for PM resistance, Schouten et al. [31] mentioned that two previously described QTLs for PM resistance co-localized with the other two cucumber clade V *MLO* genes, *CsaMLO1* and *CsaMLO11*. Fukino et al. [35] performed QTL analysis in a RIL population derived from a cross between the PM resistant genotype CS-PMR1 (an inbred line derived from the PM resistant wild cucumber accession PI 197088) and the moderately susceptible genotype Santou, a native Japanese cultivar. Of the nine detected QTLs for PM resistance, one QTL (*pm1.1*) co-localized with *CsaMLO1*, whereas another QTL (*pm6.1*) co-localized with *CsaMLO11*. The resistance associated with *pm1.1* was contributed by the allele from CS-PMR1, while the Santou allele contributed to resistance at the *pm6.1* locus.

Here we report the functional characterization of two cucumber clade V *MLO* genes, *CsaMLO1* and *CsaMLO11*. We show that heterologous overexpression of either of the genes in a tomato *mlo* mutant led to restoration of susceptibility to PM. Furthermore, we investigated the transcription profile of the three cucumber clade V *MLO* genes in various tissues, both prior to and after inoculation with the PM causing fungus *P. xanthii*. Also, we screened a set of 115 resequenced cucumber accessions in silico for potential loss of function mutations in either of the clade V *MLO* genes, and resequenced two additional cucumber genotypes with reported PM resistance QTLs in regions containing either *CsaMLO1* or *CsaMLO11*.

## Results

### Functional analysis of cucumber clade V *MLO* genes by complementation of a tomato *mlo* mutant

We amplified the clade V *MLO* genes *CsaMLO1* [Csa1M085890] and *CsaMLO11* [Csa6M292430] from cDNA of a cucumber inbred line. PCR products were of the expected sizes (1.749 bp and 1.782 bp, respectively) and sequences were identical to the reference genome of the PM susceptible genotype ‘Chinese Long 9930’ [30]. To test whether these genes are susceptibility genes, *CsaMLO1* and *CsaMLO11* were overexpressed in a tomato *mlo* mutant, which is resistant to PM due to a mutation in the *SlMLO1* gene [11]. It was expected that if *CsaMLO1* and/or *CsaMLO11* are susceptibility genes, overexpression in the tomato *mlo* mutant would lead to restoration of susceptibility to PM. After transformation, cuttings from eight (*CsaMLO1*) or seven (*CsaMLO11*) individual transformants were obtained and inoculated with *Oidium neolycopersici*, the causal agent of PM in tomato. Sporulation was observed on five of the eight *CsaMLO1* transformants and on two of the seven *CsaMLO11* transformants (Table 1).

**Table 1:** Cuttings of five out of eight *CsaMLO1* transformants and two out of seven *CsaMLO11* transformants were found to be susceptible to *Oidium neolycopersici*

Gene	Transformant	Transgene expression	PM symptoms
*CsaMLO1*	35S::*CsaMLO1*-A	2.3	+
	35S::*CsaMLO1*-B	1.3	+
	35S::*CsaMLO1*-C	1.2	+
	35S::*CsaMLO1*-D	0.8	−
	35S::*CsaMLO1*-E	0.7	−
	35S::*CsaMLO1*-F	0.4	−
	35S::*CsaMLO1*-G	0.4	+
	35S::*CsaMLO1*-H	0.1	+
*CsaMLO11*	35S::*CsaMLO11*-A	0.3	−
	35S::*CsaMLO11*-B	0.3	−
	35S::*CsaMLO11*-C	0.2	+
	35S::*CsaMLO11*-D	0.2	−
	35S::*CsaMLO11*-E	0.1	−
	35S::*CsaMLO11*-F	0.1	−
	35S::*CsaMLO11*-G	0.1	+

The tomato *Slmlo1* mutant, with a frameshift deletion in the *SlMLO1* gene [11], was transformed with either a 35S::*CsaMLO1* construct or a 35S::*CsaMLO11* construct. *CsaMLO1* or *CsaMLO11* expression in each of the primary transformants (one sample per individual transformant) was quantified relatively to housekeeping gene *SlEF-α* using qRT-PCR. Two cuttings per individual transformant were inoculated with *Oidium neolycopersici*, the causal agent of powdery mildew (PM) in tomato. Disease phenotypes were scored based on whether or not PM symptoms were visible on the leaves at 10 days post inoculation

In order to confirm the ability of *CsaMLO1* and *CsaMLO11* to restore PM susceptibility, primary transformants were self-pollinated to obtain T2 families. T2 families were obtained from two individual transformants per gene. Additionally, two T2 families were obtained from previously described *CsaMLO8* [Csa5M623470] overexpressing transformants [13]. From each T2 family, 22 to 30 plants were sown. Plants were screened by PCR for the presence of the overexpression construct. It was found that the T2 populations obtained from the *CsaMLO1* and *CsaMLO11* transformants segregated for the presence of the transgene in ratios close to 3:1, suggesting one transgene insertion site per individual primary transformant. The T2 populations obtained from (both) the *CsaMLO8* transformants showed skewed segregation patterns with either one out of 22 (T2-A) or one out of 30 (T2-B) individuals not having the transgene, suggesting multiple inserts per individual transformant.

**Fig. 1:**
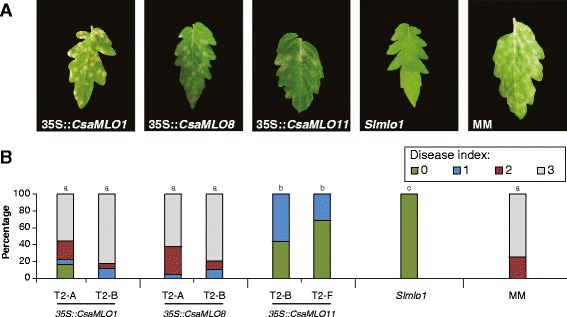
Complementation of a tomato *mlo* mutant with either *CsaMLO1*, *CsaMLO8* or *CsaMLO11*. The tomato *mlo* mutant, with a frameshift deletion in the *SlMLO1* gene [11], was transformed with either a 35S::*CsaMLO1* construct, a 35S::*CsaMLO8* construct or a 35S::*CsaMLO11* construct (Table 1). Two individual transformants per transgene were self-pollinated to obtain T2 populations. T2 plants were screened for the presence of the overexpression construct. Plants carrying an overexpression construct were inoculated with *O. neolycopersici*. **a** Representative individuals from T2 families expressing either *CsaMLO1*, *CsaMLO8* or *CsaMLO11* in the tomato *mlo* mutant, showing powdery mildew (PM) symptoms. Non-transformed tomato *slmlo1* mutant (PM resistant) and cv. Moneymaker (MM, PM susceptible) are shown as controls. **b** Disease indices were scored visually on a scale from 0 (completely resistant) to 3 (completely susceptible), as described in [11]. T2 families consisted of 16 to 29 individuals positive for the presence of the overexpression construct. Resistant and susceptible controls consisted of 12 individuals. Bars represent percentages of plants within each disease index class. Different letters above bars indicate significant differences between populations (Kruskal-Wallis test with Stepwise-Stepdown Multiple Comparisons, *P* < 0.05)

T2 families were inoculated with *O. neolycopersici* and PM symptoms were scored based on a 0–3 scale, with 0 being completely free of PM symptoms and 3 being fully infected. The non-transformed *slmlo1* mutant did not show any PM symptoms (i.e. all plants scored a disease index of 0), whereas in the susceptible control (Moneymaker) 75% of the plants scored the maximum disease index of 3, and 25% of the plants scored a disease index of 2 (Fig. 1). An overall analysis showed significant differences between the groups (Kruskal-Wallis test, *P* < 0.05). Stepdown post hoc analysis revealed that overexpression of *CsaMLO8* or *CsaMLO1* restored susceptibility completely, leading to a susceptibility level not significantly different from Moneymaker (*P* > 0.05). However, overexpression of *CsaMLO11* restored susceptibility only partially, giving disease indices between 0 and 1, which is significantly higher than the resistant control *slmlo1* (*P* < 0.05) but significantly lower than the susceptible control Moneymaker (*P* < 0.05).

Per family, five plants (positive for the presence of the overexpression construct) were randomly chosen to measure the expression of the transgene using qRT-PCR (Additional file 1). It was found that the transgene expression in both *CsaMLO8* T2 families was significantly higher than in the *CsaMLO1* T2-A family and both of the *CsaMLO11* T2 families (ANOVA with Bonferroni post hoc test, *P* < 0.05), whereas there was no significant difference in transgene expression between *CsaMLO1* and *CsaMLO11* T2 families (ANOVA with Bonferroni post hoc test,*P* > 0.05). The *CsaMLO1* T2-B family did not have a significantly different transgene expression compared to either of the other T2 families (ANOVA with Bonferroni post hoc tests,*P* > 0.05). Transgene expression was not detectable in (untransformed) susceptible Moneymaker or resistant *mlo* control plants.

### Transcript abundance profiling of cucumber clade V *MLO* genes

**Fig. 2:**
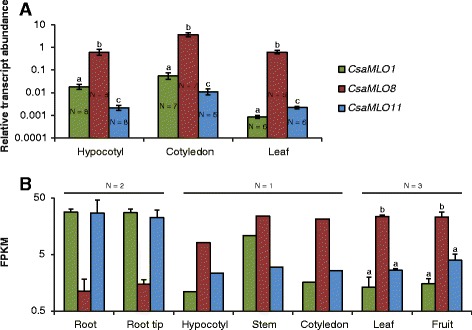
Expression profile of clade V cucumber *MLO* genes in different tissues. **a** The relative transcript abundances of *CsaMLO1*, *CsaMLO8* and *CsaMLO11* in three tissues of the PM-susceptible cucumber cultivar ‘Sheila’ (hypocotyl, cotyledon and leaf) were determined using qRT-PCR. Data were normalized, using the geometric average of the Ct values of reference genes *Ef-α* and *TIP41*. Relative transcript abundances were calculated as 2^-dCt^. Each bar shows the average transcript abundance of five to eight biological replicates on a logarithmic scale. The number of independent biological replicates per gene/tissue combination is given in the respective bars. Error bars indicate standard error of the mean. Different letters above bars indicate significant differences between genes (ANOVA with Bonferroni post hoc tests, *P* < 0.05). **b** The transcript abundances in seven tissues of cucumber (‘Chinese long’ inbred line 9930) were determined using RNA-seq. The FPKM values (Fragments Per Kilobase of transcript per Million mapped fragments) for *CsaMLO1*, *CsaMLO8* and *CsaMLO11* in each of these tissues are shown on a logarithmic scale. The amount of independent biological replicates per tissue was either one (hypocotyl, stem and cotyledon), two (root and root tip) or three (leaf and fruit). If applicable, error bars indicate the standard error of the mean. Different letters above bars indicate significant differences between genes (ANOVA with Bonferroni (for fruit tissue) or Dunnet T3 (for leaf tissue) post hoc tests, *P* < 0.05)

The relative transcript abundance of the cucumber clade V *MLO* genes *CsaMLO1*, *CsaMLO8* and *CsaMLO11* was determined in three different tissues (hypocotyl, cotyledon and leaf) of the susceptible cucumber cultivar Sheila, using qRT-PCR. It was found that in each of the three tissues the transcript abundance of *CsaMLO8* was several orders of magnitude higher than that of *CsaMLO1* and *CsaMLO11* (Fig. 2a), a difference which was found to be statistically significant (ANOVA with Bonferroni post hoc tests, *P* < 0.05). To confirm this, we re-examined a previously obtained dataset consisting of RNA-seq data obtained from a variety of cucumber tissues from the reference cultivar ‘Chinese Long 9930’. This showed that in all examined aerial tissues (hypocotyl, stem, cotyledon, leaf and fruit tissue) *CsaMLO8* was higher expressed (on average ca. tenfold) compared to either *CsaMLO1* or *CsaMLO11*, although in stem tissue *CsaMLO1* also appeared to be rather highly expressed. For the aerial tissues for which data on more than one biological replicate was available (leaf and fruit tissue), the observed difference in expression was found to be statistically significantly (ANOVA with Bonferroni post hoc tests, *P* < 0.05). In root and root tip tissue however, both *CsaMLO1* and *CsaMLO11* were highly expressed, whereas *CsaMLO8* was lowly expressed (Fig. 2b), although this difference was not found to be statistically significant (ANOVA, *P* > 0.05). For comparison we also examined the expression of the *A. thaliana* Clade V *MLO* genes (*AtMLO2*, *AtMLO6* and *AtMLO12*) in a publicly available RNA-seq dataset of *A. thaliana* tissues [36]. We found that *AtMLO2* expression was much higher than *AtMLO6* or *AtMLO12* expression in all four sampled tissues (root, leaf, flower and fruit), although the difference in roots was smaller than in the other tissues (Additional file 2).

**Fig. 3:**
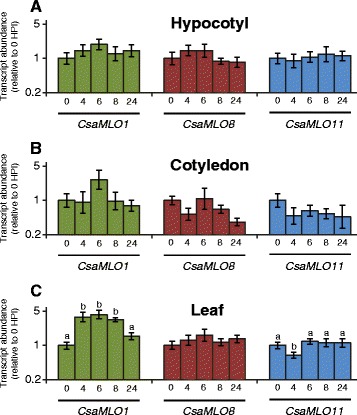
Relative expression level of clade V *MLO* genes in different cucumber tissues in response to *Podosphaera xanthii* inoculation. Relative transcript abundances in three tissues (**a**) hypocotyl, **b** cotyledon and **c** leaf) of PM-susceptible cucumber genotype ‘Sheila’ before and at 4, 6, 8 and 24 h post inoculation with *P. xanthii* were determined using qRT-PCR. Data were normalized relative to the geometric average of the Ct values of reference genes *Ef-α* and *TIP41*, and subsequently normalized relative to the average dCt value at 0 hpi for each gene/tissue combination. Relative transcript abundances were calculated as 2^-ddCt^. Each bar shows the relative expression of four to eight biological replicates on a logarithmic scale. Error bars indicate standard error of the mean. Different letters above bars indicate significant differences between time points (ANOVA with Bonferroni post hoc tests, *P* < 0.05)

Subsequently we investigated the expression profile in cucumber tissues inoculated with *P. xanthii*. Samples were taken of cucumber hypocotyl, cotyledon and leaf tissue prior to and at four, six, eight and 24 h post inoculation. The relative expression of *CsaMLO1*, *CsaMLO8* and *CsaMLO11* in those samples was determined using qRT-PCR (Fig. 3). We found that in leaf tissue there were significant differences in *CsaMLO1* transcript abundance between time points (ANOVA, *P* < 0.05). At four, 6 and 8 h post inoculation *CsaMLO1* transcript abundance was significantly higher compared to the transcript abundance prior to inoculation (Bonferroni post hoc tests, *P* < 0.05). This induction of *CsaMLO1* was not significant anymore at 24 h after inoculation (Bonferroni post hoc test, *P* > 0.05). *CsaMLO11* was significantly downregulated at 4 h post inoculation compared to the transcript abundance prior to inoculation (ANOVA with Bonferroni post hoc test, *P* < 0.05). In cotyledon or hypocotyl tissue there were no significant differences of Clade V *MLO* gene transcript abundance between any of the time-points (ANOVA, *P* > 0.05).

As these results were in conflict with the finding in [13] that *CsaMLO8* transcription was upregulated upon PM inoculation, the experiment was repeated independently, with samples of cucumber hypocotyl and leaf tissue prior to and at 4, 6, 8, 12 and 24 h post inoculation. The relative expression of *CsaMLO8* in those samples was determined using qRT-PCR (Additional file 3). No significant differences in *CsaMLO8* transcript abundance were observed in any of the time points compared to the transcript abundance prior to inoculation (ANOVA, *P* > 0.05).

### Screening of sequenced cucumber germplasm for potential *CsaMLO1* or *CsaMLO11* mutants

We anticipated that if *CsaMLO1* and *CsaMLO11* are susceptibility genes towards PM in cucumber, loss-of-function mutations in those genes, leading to resistance, would have been selected for in cucumber germplasm. As Qi et al. published resequencing data of a collection of 115 divergent cucumber genotypes [37], we decided to screen these data for potential loss-of-function mutations in either *CsaMLO1* or *CsaMLO11*. Complete lists of detected SNPs and indels in the 115 lines were downloaded from the Cucurbit Genomics Database [38], and filtered for the chromosomal locations of *CsaMLO1* and *CsaMLO11*. Additional files 4 and 5 give an overview of all the detected SNPs and indels in *CsaMLO1* and *CsaMLO11* regions, respectively. The lists of SNPs/indels were manually curated to obtain variants in coding regions of the genes, with an effect on the predicted amino acid sequence. For *CsaMLO11* we observed no SNPs or indels with an effect on the predicted protein. For *CsaMLO1* three SNPs (and no indels) were found with an effect on the predicted amino acid sequence (Table 2). The first substitution (V170G) was in an amino acid residue conserved to be either a Valine, an Isoleucine or a Leucine in other clade V MLO proteins with a proven function as susceptibility gene (Additional file 6). The other two detected substitutions (V472I and V557I) were in non-conserved regions.

**Table 2:** Non-synonymous SNPs detected in the *CsaMLO1* coding sequence of 115 resequenced cucumber accessions

Chromosome	Chromosome position	Exon number	Nucleotide substitution	Amino acid substitution
Chr1	8,163,552	Exon 4	T - > G	V170G
Chr1	8,159,763	Exon 14	G - > A	V472I
Chr1	8,159,508	Exon 14	G - > A	V557I

### Resequencing of cucumber genotypes CS-PMR1 and Santou to find potential *CsaMLO1* or *CsaMLO11* mutants

As it was mentioned that two previously reported QTLs for PM resistance co-localize with the genomic positions of *CsaMLO1* and *CsaMLO11* [31], we hypothesized that the sources of these resistances might have mutant alleles of *CsaMLO1* and/or *CsaMLO11*, causal for the observed resistance. In order to test this hypothesis, we isolated DNA from leaves of genotypes CS-PMR1 and cv. Santou, the parental genotypes used for the QTL analysis [35], and performed whole genome sequencing (WGS). We aligned the obtained reads to the reference genome (Chinese long 9930 [30]) and identified SNPs and small indels in the genomic regions of *CsaMLO1* and *CsaMLO11* (Additional files 7, 8 and 9). In the genomic region of *CsaMLO1* we identified 23 SNPs and indels in CS-PMR1 and four SNPs and indels in Santou, but none of these had an effect on the predicted encoded protein. Similarly we identified 23 SNPs and indels in the genomic region of *CsaMLO11* in CS-PMR1, neither of which had an effect on the predicted encoded protein. The *CsaMLO11* sequence of Santou was found to be identical to that of the reference genome.

In addition to calling SNPs and indels we identified two regions in genotype CS-PMR1, in intron 6 of *CsaMLO1* and intron 12 of *CsaMLO11* respectively, where we observed a local low read coverage combined with flanking read pairs with insert sizes deviating from the average or for which one of the mates in the read pair could not be mapped (Additional files 7 and 8). As this can be indicative for larger structural variations that are harder to characterize with short read sequencing, we amplified these regions from DNA of CS-PMR1 and sequenced the PCR products by Sanger sequencing. Compared to the reference genome, a 10 bp deletion and a 23 bp insertion in intron 6 of *CsaMLO1* (Additional file 10A), and a 231 bp deletion in intron 12 of *CsaMLO11* (Additional file 10D) were found in genotype CS-PMR1. To verify whether these large indels in intron 6 of *CsaMLO1* and intron 12 of *CsaMLO11* have any effect on the splicing of the genes, we also amplified and sequenced the corresponding regions from cDNA of CS-PMR1 and Santou by PCR, and found that there was no observable difference in PCR product size or sequence for either *CsaMLO1* (Additional file 10B-C) or *CsaMLO11* (Additional file 10E-F).

As we anticipated that the resistances of genotypes Santou and CS-PMR1 could also be caused by a difference in *CsaMLO1* or *CsaMLO11* expression rather than a difference in encoded protein sequence, we determined the relative transcript abundances of *CsaMLO1* and *CsaMLO11* in leaf tissue of both genotypes by qRT-PCR, using the susceptible genotype Sheila as a control (Additional file 11). Although the relative transcript abundance of both genes was found to be slightly higher in CS-PMR1 compared to the other two genotypes, these differences were found to be not statistically significant for either *CsaMLO1* (ANOVA, *P* = 0.156) or *CsaMLO11* (ANOVA, *P* = 0.239).

## Discussion

### Overexpression of all three cucumber clade V MLO genes restores PM susceptibility in a tomato *mlo* mutant

Previously, it has been shown that loss of susceptibility towards PM causing fungi due to mutations in *MLO* genes can be restored by overexpression of functional *MLO* genes, both by cloning an *MLO* gene from a susceptible individual from the same plant species [10–12] as well as by heterologous expression of clade V *MLO* genes from other dicot species [13, 15, 39], and even by heterologous expression of clade IV *MLO* genes from a monocot species [29]. This shows that even though there are considerable differences in amino acid sequence between MLO proteins, they are functionally conserved between plant species. This enabled us to study the function of cucumber clade V *MLO* genes by heterologous expression in the tomato *slmlo1* mutant background. We have shown that overexpression of either of the three clade V *CsaMLO* genes restored susceptibility to *O. neolycopersici*, the PM causing fungus in tomato, albeit to a different extent. Overexpression of either *CsaMLO1* or *CsaMLO8* resulted in full restoration of PM susceptibility towards wild-type levels. Overexpression of *CsaMLO11* on the other hand only partially complemented loss of *SlMLO1* function (Fig. 1). This shows that, at least to some extent, all three genes are functionally conserved.

Overexpression of *CsaMLO11* appeared to be less efficient in restoring PM susceptibility compared to the other *CsaMLO* genes (Fig. 1). Although the proteins encoded by each of the three genes are not identical, they are highly similar to one another and to clade V *MLO* genes of other species (Additional file 6). It appears difficult to attribute the lower efficiency of *CsaMLO11* compared to the other *CsaMLO* genes in restoring PM susceptibility to a particular difference in amino acid sequence.

### *CsaMLO8* is the major clade V *MLO* gene in aerial tissues, whereas *CsaMLO1* and *CsaMLO11* are the major clade V genes in roots

Susceptibility genes are defined as those genes that facilitate infection and support compatibility to plant pathogens [40]. Within the *MLO* gene family, homologs from clade IV (in monocotyledonous species) and V (in dicotyledonous species) have been found to be susceptibility genes [24]. However, not all clade V *MLO* genes in all dicotyledonous plant species have been found to be S-genes. For instance, in grapevine (*Vitis vinifera*) it was found that there are four clade V *MLO* genes. Silencing of one of them (*VvMLO7*) by transformation with RNAi constructs led to gain of PM resistance, whereas silencing of two other homologs only increases resistance when *VvMLO7* was already silenced. Silencing of the fourth homolog did not contribute to resistance at all [16]. This unequal genetic redundancy was previously also observed in *Arabidopsis* with one major *MLO* S-gene (*AtMLO2*) and two minor *MLO S-*genes [10]; and in tomato, with one major *MLO* S-gene (*SlMLO1*), two minor *MLO* S-genes and one clade V *MLO* homolog which does not seem to play a role in PM susceptibility [41].

We have shown, using qRT-PCR data (Fig. 2a) and RNA-seq data (Fig. 2b), that in aerial cucumber tissues (hypocotyl, stem, cotyledon, leaf and fruit tissue) *CsaMLO8* is several folds higher expressed than the other cucumber clade V *MLO* genes, *CsaMLO1* and *CsaMLO11*. This is reminiscent of the findings in tomato by Zheng et al. [41], who showed that the major clade V *MLO* gene, *SlMLO1*, is much higher expressed than the other clade V homologs in case of absence of PM. Interestingly, they showed that silencing of *SlMLO1* by transformation with an RNAi construct led to gain of resistance, whereas silencing of the other *MLO* homologs did not. Indeed, a natural *slmlo1* loss-of-function mutant had previously been characterised to be resistant to PM [11], even though the other clade V *MLO* genes were presumably still intact. Additionally, we have shown that in a publicly available *Arabidopsis* RNA-seq dataset one clade V *MLO* homolog, *AtMLO2*, is much higher expressed than the other clade V homologs, *AtMLO6* and *AtMLO12* (Additional file 2). Previously it has been shown that loss-of-function mutations in *AtMLO2*, but not in *AtMLO6* or *AtMLO12* lead to (partial) resistance against PM, although double *AtMLO2/6* or *AtMLO2/12* and triple *AtMLO2/6/12* mutants showed even higher levels of resistance [10].

Recently, researchers investigated the expression pattern of *MLO* genes in a large number of tissues from *Arabidopsis* and rice, based on microarray data [42]. The data presented there for the *Arabidopsis MLO* genes are in agreement with our findings (Additional file 2), as they report that in most tissues the major *MLO* S-gene *AtMLO2* is much higher expressed than the other two clade V *MLO* genes [42]. Rice has two clade IV *MLO* genes, *OsMLO3* and *OsMLO6* [25]*,* it was found that in most rice tissues *OsMLO3* transcription was much higher than *OsMLO6* transcription [42]. Based on this finding, the authors conclude that *OsMLO3* is likely to be the major clade IV *MLO* in rice, rather than *OsMLO6*.

Taken together, this suggests that loss-of-function mutations in the most abundantly expressed *MLO* gene have a large effect compared to loss-of-function mutations in the less abundantly expressed genes. This would imply that in cucumber, *CsaMLO8* would be the major clade V *MLO* gene concerning PM susceptibility, comparable in function to e.g. *SlMLO1* in tomato and *AtMLO2* in *Arabidopsis*. We postulate that differences in transcription efficiency between different clade V *MLO* genes in a species are the main reason for the observed unequal genetic redundancy, and that characterization of the relative transcript abundances of clade V *MLO* genes in a species may help identify the major *MLO* gene regarding susceptibility. To our knowledge, it has not been attempted in either *Arabidopsis* or tomato to express the minor clade V *MLO* genes under a strong constitutive promoter. On the basis of our results, we would expect that overexpression of e.g. *AtMLO6* or *AtMLO12* in *Atmlo2* background would be sufficient for restoration of susceptibility, if it were to be true that transcript abundance rather than differences in amino acid sequence determine which clade V *MLO* gene is the major S-gene.

Contrastingly, RNA-seq results show that *CsaMLO1* and *CsaMLO11* are highly expressed in root tissue, whereas *CsaMLO8* is not (Fig. 1b). Interestingly, all three *Arabidopsis* Clade V *MLO* genes were also found to be highly expressed in root tissue (Additional file 2). As PM causing fungi are foliar pathogens, which do not infect roots, the finding that several clade V *MLO* genes in cucumber and *Arabidopsis* are highly expressed in roots will probably not have much consequence on the interaction of plants with PM causing fungi. However, we should note that it is likely that *CsaMLO1* and *CsaMLO11* fulfil an important, yet unknown role in cucumber roots.

Loss-of-function mutations in *HvMLO* in barley and *AtMLO2*/*AtMLO6* in *Arabidopsis* lead to increased susceptibility to necrotrophic and hemibiotrophic pathogens such as leaf spot blotch disease caused by *Bipolaris sorokiniana* [43], rice blast on barley caused by *Magnaporthe grisea* [44], leaf spot disease caused by *Alternaria* spp. and late blight caused by *Phytophthora infestans* [10]. This suggests that *MLO*-genes can in some cases contribute to resistance to necrotrophic and hemibiotrophic pathogens, in contrast to their role as susceptibility gene for (biotrophic) PM causing fungi. Therefore, it might be interesting to study the effect of loss-of-function mutations in the root-expressed *CsaMLO1* and *CsaMLO11* on the interaction with necrotrophic, soil-borne cucumber pathogens such as vascular wilt caused by the necrotrophic fungus *Fusarium oxysporum* f. sp. *cucumerinum* or root rot caused by the necrotrophic oomycete *Pythium* spp. Furthermore, as it is known that barley *mlo* mutants are less efficiently colonized by mutualistic arbuscular mycorrhiza fungi [45], it could also be interesting to see the effect of *CsaMLO1* and *CsaMLO11* loss-of-function mutations on the mutualistic interaction with arbuscular mycorrhiza fungi in cucumber.

### *CsaMLO1* expression is induced upon PM inoculation, whereas *CsaMLO8* and *CsaMLO11* are not

For several plant species it has been shown that the expression of *MLO* susceptibility genes is induced upon inoculation with PM causing fungi (e.g. [46–48]), potentially due to the fungus actively upregulating the expression of those genes to induce susceptibility. It was previously reported based on RNA-seq data of *P. xanthii* inoculated cucumber leaves that *CsaMLO1*, but not *CsaMLO8* or *CsaMLO11* was upregulated in leaves in response to the inoculation, showing a ca. 3.5 fold upregulation in expression 8 h post inoculation [31]. Figure 3c confirms this finding, since we have found significant (ca. four-fold) upregulation of *CsaMLO1* expression in leaves at 4, 6 and 8 h after inoculation with *P*. *xanthii*. As upregulation of *MLO* gene expression due to inoculation with PM causing fungi is often regarded as putative evidence for a role as susceptibility gene, one might argue that this suggests that *CsaMLO1* is a functional susceptibility gene, and *CsaMLO8* or *CsaMLO11* are not. However we would like to point out that even though *CsaMLO1* expression in leaves is significantly induced upon PM inoculation relative to the expression before inoculation (Fig. 3c), the expression of *CsaMLO1* before inoculation is much lower than that of *CsaMLO8* which is constitutively higher expressed (Fig. 2), so consequently even after inoculation the transcript abundance of *CsaMLO8* is still higher than that of *CsaMLO1*.

Previously we observed that inoculation of cucumber with *P. xanthii* led to upregulation of *CsaMLO8* in hypocotyl tissue, but not in leaf or cotyledon [13]. To our surprise we could not reproduce this result in our experiments described here, even though using the same cucumber genotype, *P. xanthii* isolate, climatic conditions and inoculation protocol, and using the same qRT-PCR primers and protocol (Fig. 3a). Even though we did observe a small induction of *CsaMLO8* expression in hypocotyl at 4 and 6 h post inoculation, differences in *CsaMLO8* transcript abundance in hypocotyl tissue between time points were far from significant (ANOVA, *P* = 0.389). It should be noted that the variation in transcript abundances between different biological replicates is quite high, both in our experiments described here and in our previously published results. As the current experiment has a larger sample size (four to eight independent biological replicates per time point instead of three independent biological replicates in [13]) and was repeated with similar results (Additional file 3), we conclude that upregulation of *CsaMLO8* in hypocotyl tissue [13] was probably an artefact caused by a low number of biological replicates.

We previously described that a loss-of-function mutant allele of *CsaMLO8* leads to hypocotyl-specific resistance towards PM, with partial resistance in leaf tissue, and attributed this tissue specificity to the supposed tissue-specific upregulation of *CsaMLO8* [13]. Now that we have shown that *CsaMLO8* is in fact not upregulated in hypocotyl tissue, we have to come up with a different explanation for the observed tissue specificity of *Csamlo8*-based resistance. It is in this sense interesting to note that *CsaMLO1* basal expression is very low (Fig. 2) whereas it is induced upon PM inoculation in leaf tissue but not in hypocotyl tissue (Fig. 3). Assuming that both *CsaMLO1* and *CsaMLO8* are functional susceptibility genes, we can hypothesize that in a *Csamlo8* loss-of-function mutant, which expresses functional *CsaMLO1*, there is hardly any expression of a functional *MLO* susceptibility gene in hypocotyl tissue, whereas there is induced expression of *CsaMLO1* in leaf tissue, resulting in complete resistance in hypocotyl tissue and only partial resistance in leaf tissue. If this would be true, a double *Csamlo1*/*Csamlo8* loss-of-function mutant would be expected to have complete resistance in both leaf and hypocotyl tissue.

### No putative loss-of-function *CsaMLO1* or *CsaMLO11* mutants could be identified in resequenced cucumber germplasm

As loss-of-function mutations in functional susceptibility genes can lead to durable resistance [40], it would be worthwhile to obtain cucumber lines with mutations in clade V *MLO* genes. It has previously been described that a natural mutant allele in *CsaMLO8*, caused by insertion of a retrotransposable element, leads to partial resistance to *P. xanthii*. This mutant allele has a rather high frequency in breeding material, probably because of its beneficial effect on PM resistance [13]. Furthermore, several other mutant alleles of *CsaMLO8* were identified in resistant cucumber genotypes [33]. It is therefore reasonable to assume that if loss-of-function mutations in *CsaMLO1* and/or *CsaMLO11* would contribute to PM resistance, they also would have been selected for during cucumber breeding. Therefore we decided to screen a publicly available dataset of SNPs and indels in a collection of 115 cucumber genotypes [37] for putative loss-of-function alleles in the coding regions of *CsaMLO1* and *CsaMLO11* (Additional files 4 and 5). We did not find any evidence for variant alleles with a large effect on the amino acid sequence in either of the genes (e.g. a SNP leading to gain of an early stop codon or a frameshift indel), although we observed three SNPs in *CsaMLO1* leading to amino acid substitutions (Table 2). Of these three SNPs, two were predicted to cause an amino acid substitution from valine to isoleucine, two amino acid residues with very similar physiochemical properties. Furthermore, those amino acid residues were in the C-terminal domain of the CsaMLO1 protein, a region which is not conserved compared to other clade V MLO proteins (Additional file 6). A third SNP was predicted to lead to a substitution of the 170th amino acid residue, a valine, into a glycine residue, at a location conserved to be either a valine, a leucine or an isoleucine in other clade V MLO proteins (Additional file 6). As glycine and valine are both relatively small, aliphatic, non-polar amino acids, this substitution can be considered a rather conservative mutation. Without further evidence it does not seem very likely that this SNP represents a loss-of-function allele of *CsaMLO1*. In conclusion, we did not find strong evidence in this dataset for possible loss-of-function alleles of *CsaMLO1* and/or *CsaMLO11*, although it should be noted that by focussing on SNPs and indels we could have overlooked mutant alleles that are harder to find by short-read resequencing, such as the transposable element characterised in *CsaMLO8*, which can have a profound effect on the function of the genes.

In another approach to try to identify possible mutant alleles of *CsaMLO1* or *CsaMLO11* we resequenced two additional cucumber genotypes, CS-PMR1 and cv. Santou, which were previously mentioned to have QTLs for PM resistance colocalizing with the genomic positions of *CsaMLO1* and *CsaMLO11* [31, 35]. Although we identified several SNPs, indels and structural variations at the *CsaMLO1* and *CsaMLO11* loci, especially in the more PM resistant genotype CS-PMR1, none of these are predicted to lead to any change in the encoded CsaMLO1 or CsaMLO11 proteins (Additional files 7, 8, 9 and 10). In addition, we verified whether the transcript abundances of *CsaMLO1* and *CsaMLO11* were different between these cucumber genotypes (Additional file 11), but we concluded that there were no significant differences among them. Therefore, we concluded that the observed resistance by Fukino et al. [35] is likely caused by other genes rather than *CsaMLO1* or *CsaMLO11*.

The fact that we could not find convincing loss-of-function alleles of *CsaMLO1* or *CsaMLO11* in a diverse panel of cucumber genotypes implies that loss-of-function mutations in either of these genes have apparently not been selected for in cucumber breeding. An explanation for this finding could be that *Csamlo1* and *Csamlo11* knockout mutations could have only a small, additive effect on PM resistance in *Csamlo8* mutant background, and not have any effect in *CsaMLO8* background, comparable to the situation in *Arabidopsis* [10]. Furthermore it could be possible that loss-of-function mutations in *CsaMLO1* and *CsaMLO11* have pleiotropic effects on plant fitness, and are therefore selected against.

It would in our opinion be interesting to study the effect of knock-out mutants of *CsaMLO1* and *CsaMLO11*, for instance by targeted mutation using the increasingly popular CRISPR-Cas9 technology [49], such as was already done in the bread wheat *TaMLO-A1* gene [50]. This might lead to a new, durable source of PM resistance in cucumber, especially when combined with the already existing *Csamlo8* partial resistance [13].

## Conclusions

In this study we analysed the role of cucumber clade V *MLO* genes in susceptibility to PM. We showed by means of heterologous overexpression of the cucumber *MLO* genes *CsaMLO1*, *CsaMLO8* and *CsaMLO11* that all three genes are able to restore susceptibility in *mlo* tomato, although the effect of *CsaMLO11* overexpression was weaker compared to *CsaMLO1* or *CsaMLO8* overexpression. Additionally, we studied the transcription levels of these genes in different tissues of cucumber, both with and without inoculation with a PM causing fungus, *P. xanthii*, showing that *CsaMLO8* is higher expressed compared to *CsaMLO1* and *CsaMLO11* in all aerial tissues, although *CsaMLO1* expression in leaves is induced by inoculation with *P. xanthii*. We discuss that *CsaMLO8* is therefore likely to be the major clade V *MLO* gene in cucumber concerning PM susceptibility, with a potential minor role for *CsaMLO1* and *CsaMLO11*, comparable to earlier findings for *mlo* genes in *Arabidopsis* and tomato. In roots, however, *CsaMLO1* and *CsaMLO11* appeared to be much higher expressed, which might have implications on the interactions of cucumber with root pathogens or beneficial microbes. No potential natural loss-of-function mutations in either *CsaMLO1* or *CsaMLO11* have been found so far. It would be interesting to generate *Csamlo1* and *Csamlo11* mutants, for instance by CRISPR-Cas9 technology, to investigate whether such mutations have an added effect on top of PM resistance caused by *Csamlo8*.

## Methods

### Cloning of *CsaMLO1* and *CsaMLO11*

A homozygous cucumber breeding line derived from a parental line of cv. Anaxo was grown in a greenhouse in Wageningen, the Netherlands. Growing conditions were 20 °C (day) and 16 °C (night), with a 16 h/8 h day/night cycle, and a relative humidity of 70%. RNA isolation and cDNA synthesis were performed as previously described [13].

The coding sequence of *CsaMLO1* was amplified from cDNA with primers 5′-caccTTCCTTCCACACCCCTAAGA-3′ (Forward) and 5′-TGAATGGTGTAAACGAGATTGC-3′ (Reverse). As template 50 ng cDNA was used in a 50 μl reaction using Advantage 2 polymerase (Takara Bio, U.S.A.). Cycling conditions were: 1 min initial denaturation at 95 °C, followed by 35 cycles of 30 s denaturation at 95 °C and 3 min annealing and extension at 68 °C. Reactions were finished by 3 min incubation at 68 °C. The PCR product was subsequently diluted 100 times, and used as template for a 50 μl reaction using Phusion high-fidelity polymerase (ThermoFisher Scientific, U.S.A.). Cycling conditions were: 30 s initial denaturation at 98 °C, followed by 25 cycles of 20 s denaturation at 98 °C, 30 s annealing at 55 °C, and 30 s extension at 72 °C. Reactions were finished by 10 min incubation at 72 °C.

The coding sequence of *CsaMLO11* was amplified from cDNA as previously described [13], using primers 5′-caccTTTGTTTCCCTACGCGTTCT-3′ (Forward) and 5′-TATACCAACCCCCAACCTCA-3′ (Reverse).

Cloning of *CsaMLO1* and *CsaMLO11* PCR products, through the Gateway-compatible vector pENTR/D-TOPO (ThermoFisher Scientific, U.S.A.) to binary vector pK7WG2, which harbours the constitutively active 35S Cauliflower Mosaic Virus promotor and the *nptII* selectable marker gene for kanamycin resistance [51], was done as previously described [13].

### Complementation of *ol-2* tomato with cucumber *MLO* genes

Cotyledon explants of *ol-2* mutant tomato seedlings were transformed with *CsaMLO1* and *CsaMLO11* overexpression constructs as previously described [13]. The *ol-2* mutant carries a loss-of-function mutation in the *SlMLO1* gene [11]. Obtained tomato transformants were assessed for presence of the transgenes by PCR using the same primers as used for the cloning of *CsaMLO1* and *CsaMLO11* (see above) and for presence of the *nptII* marker gene with primers 5′-GAAGGGACTGGCTGCTATTG-3′ (*nptII* forward) and 5′-AATATCACGGGTAGCCAACG-3′ (*nptII* reverse). For each of the two transformations with a different construct, seven (*CsaMLO11*) or eight (*CsaMLO1*) independent transgenic plants were selected, and were assessed for transgene expression by qRT-PCR using primer pairs specific for *CsaMLO1*: 5′-TGAAAGTTTCCGGCGGAGTT-3′ (*CsaMLO1-*Forward) and 5′-AGGAAGCTTTACCCTTGGCG-3′ (*CsaMLO1-*Reverse) or specific for *CsaMLO11*: 5′-GCGACGGCGTTGAGAAATTG-3′ (*CsaMLO11-*Forward) and 5′-GGGTGACAAGTGGTGGGAGG-3′ (*CsaMLO11-*Reverse). As housekeeping gene for normalization of *CsaMLO1* or *CsaMLO11* expression in tomato, *SlEF-α* was used, with primer pair 5′-ATTGGAAACGGATATGCCCCT-3′ (SlEF-α forward) and 5′-TCCTTACCTGAACGCCTGTCA-3′ (SlEF-α reverse). qRT-PCR was performed as previously described [13].

### Evaluation of PM resistance of *CsaMLO* overexpressing *ol-2* tomato

From each of the individual transformants of both *CsaMLO1* and *CsaMLO11* overexpressing *ol-2* plants, two cuttings were inoculated with an isolate of *Oidium neolycopersici* maintained on susceptible tomato plants in a climate chamber in Wageningen, the Netherlands, as previously described [13].

For two individual T1 transformants per overexpression construct, seeds of self-pollinated plants were harvested. In principle the T1 transformants with highest transgene expression were chosen for generation of T2 families, although as the *CsaMLO11* transformant with the highest expression did not give viable seeds another T1 transformant with enough viable seeds was randomly selected. In addition, seeds of two self-pollinated *CsaMLO8* overexpressing plants described in [13] were harvested. Twenty-two to thirty seeds of each of the six T2 families were sown on soil, plantlets were assayed for presence of the *nptII* marker gene by PCR as described above. As susceptible control, 12 seeds of cultivar Moneymaker (MM) were sown. As resistant control, 12 seeds of resistant mutant line *ol-2*, the background used for the complementation, were sown. For five plants per T2 family and for five plants of the susceptible and resistant controls, leaf samples were harvested and immediately frozen in liquid nitrogen. Transgene expression efficiency in these plants was determined by means of qRT-PCR using primers and conditions as described above. To analyse differences in transgene expression efficiency between the T2 families a one-way ANOVA test was performed on the dCt values, followed by Dunnet’s T3 post hoc tests. Homogeneity of the variances was tested using Levene’s test. All statistical analyses were performed using SPSS v23 software (IBM). T2 families and controls were inoculated with *O. neolycopersici* as described above. The susceptibility/resistance of plants was scored after two weeks on a 0–3 scale as described earlier [11]. Differences in PM susceptibility between the T2 families and controls were analysed with a nonparametric Kruskal-Wallis test followed by Stepwise Stepdown Multiple Comparison post hoc tests to identify homogeneous subsets, using SPSS v23 software (IBM).

### Expression analysis of *MLO* genes in cucumber using qRT-PCR

For expression analysis of *MLO* genes in PM-inoculated cucumber tissues, PM susceptible cucumber cultivar ‘Sheila’ was grown and inoculated with a *P. xanthii* isolate as previously described [13]. Prior to inoculation and at 4, 6, 8 and 24 h post inoculation (hpi), from eight individual plants per time point hypocotyl, cotyledon and (first) true leaf samples were harvested separately, and were immediately frozen in liquid nitrogen. RNA isolation, cDNA synthesis and qRT-PCR were performed as previously described [13]. For quantification of *CsaMLO1* and *CsaMLO11* expression, respectively, we used the primer sequences *CsaMLO1-*Forward and *CsaMLO1*-Reverse and *CsaMLO11-*Forward and *CsaMLO11-*Reverse, as described above. Primer pairs specific for the cucumber housekeeping genes *TIP41* and *EF-α*, as described by Warzybok et al. [52], were used for normalization of expression.

Samples for which the difference in Ct value between the technical replicates was larger than 1.0, or for which one or both of the technical replicates did not reach the detection threshold were excluded from the analysis. Ct values per sample were normalised by subtracting the geometric mean of the Ct values for the two housekeeping genes, giving deltaCt, abbreviated as dCt. In the time series of inoculated cucumber tissues, dCt values were subsequently normalized by the average dCt value for each gene/tissue combination at 0 hpi, giving ddCt. Averages and standard errors of ddCt values were calculated over four to eight biological replicates per gene/tissue/time point combination. Normality of ddCt distributions was tested using Shapiro-Wilk tests (*P* > 0.05). Differences in ddCt value between time points were analysed with ANOVA tests. Homogeneity of variances were tested using Levene’s test. If ANOVA tests showed a significant effect of time points (*P* < 0.05), Bonferroni post hoc tests were performed to analyse which time points were significantly different from one another. All statistical analyses were performed using SPSS v23 software (IBM). Relative transcript abundances were calculated as 2^-ddCt^.

In a second experiment, PM susceptible cucumber cultivar ‘Sheila’ was grown and inoculated with *P. xanthii* under the same conditions as described above. Samples of leaf and hypocotyl tissue were harvested prior to inoculation and at 4, 6, 8, 12 and 24 h post inoculation (hpi), from eight individual plants per time point. Total RNA was isolated using a phenol-based protocol described by [53]. cDNA synthesis and qRT-PCR were performed as described above.

In another experiment, cucumber genotypes ‘Sheila’, ‘Santou’ and ‘CS-PMR1’ were grown in a greenhouse in Wageningen, the Netherlands. Five weeks post seeding, leaf samples were harvested and were immediately frozen in liquid nitrogen. Total RNA was isolated using the RNeasy plant mini kit (Qiagen, Germany). cDNA was synthesised using 500 ng of RNA samples with the iScript cDNA Synthesis Kit (Bio-Rad Laboratories, U.S.A.). Before use in qRT-PCR, cDNA samples were diluted 2-fold. To quantify the expression of *CsaMLO1* and *CsaMLO11*, qRT-PCR was performed with conditions and subsequent data analysis as described above, normalizing dCt values using the average dCt value of the ‘Sheila’ samples.

### Analysis of *MLO* data in RNA-seq datasets

Cucumber genotype ‘Chinese long’ inbred line 9930 was cultivated under standard greenhouse conditions (20 °C (day) and 16 °C (night), with a 16 h/8 h day/night cycle, and a relative humidity of 70%). Separate samples of roots, root tips, hypocotyls, cotyledons, stems, leaves and fruit were harvested and immediately frozen in liquid nitrogen, using one (hypocotyls, cotyledons and stems), two (roots and root tips) or three (leafs and fruit) individual samples per tissue. Material was sent to KeyGene B.V., The Netherlands, for RNA-seq. Total RNA from each sample was isolated using the RNeasy plant mini kit (Qiagen, Germany). Subsequently, RNA-seq libraries were made following the TruSeq RNA Sample Preparation v2 Guide protocol. After concentration measurement by qPCR (LightCycler 480; Roche), PhiX (~0.6%) was spiked as a control according to the manufacturer’s recommendations. The libraries were pooled, and sequenced using a Illumina HiSeq 2000 sequencer. The resulting reads were sorted into single fasta files per sample based on the sample tag sequences. The obtained read length was approximately 100 nt at a minimal read length of 36 nt. Reads were aligned to the reference genome (‘Chinese long’ inbred line 9930, version 2 [30]). The transcript abundance per sample was assessed on the basis of the number of sequenced fragments, normalised by the length of the coding sequence of the gene, per million of total reads sequenced (fragments per kilobase of transcript per million sequenced reads, FPKM). We extracted FPKM values per sample from the total dataset for each of the three clade V *MLO* genes, using Excel. For samples with more than one biological replicate, differences in FPKM values were analysed with ANOVA tests, followed by Bonferroni post hoc tests if variance was homogeneous, or Dunnet T3 post hoc tests if variance was not homogeneous. Homogeneity of variances was tested using Levene’s test. All statistical analyses were performed using SPSS v23 software (IBM).

RNA-seq data for Arabidopsis were analysed using the Gene Expression Atlas from EMBL-EBI [54]. Baseline expression values in Arabidopsis tissues as quantified by [36] were filtered for clade V *MLO* genes *AtMLO2* [AT1G11310], *AtMLO6* [AT1G61560] and *AtMLO12* [AT2G39200] and downloaded as a tabular file.

### In silico screening of *MLO* sequence variants in 115 lines

Total lists of SNPs and indels identified in 115 cucumber genotypes by [37] were downloaded from the Cucurbit Genomics Database [38]. SNPs and indels were filtered by Excel based on the genomic positions of *CsaMLO1* (Chr1: 8,159,427.8,165,253, negative strand) or *CsaMLO11* (Chr6: 14,120,024.14,125,039, positive strand). Sequence variants were manually annotated based on their genomic location to see whether they were located in introns or exons, when they were located in exons the effect on the coding sequence was scored using CLC Main Workbench v. 7.6.4.

### Resequencing of CS-PMR1 and Santou

Seeds of cucumber genotypes CS-PMR1 and Santou were ordered from the Genetic Resources Center, NARO (National Agriculture and Food Research Organization), Japan. Young leaves of both genotypes were harvested and immediately frozen in liquid nitrogen. DNA was isolated from leaves as described by [55]. Total DNA was send for library preparation and whole genome sequencing with an average coverage of 25 reads per base pair using Illumina Hiseq PE150 technology with insert size of 350 bp, by Novogene Company Limited, Hong Kong, People’s Republic of China. Resulting reads were aligned to the cucumber reference genome (‘Chinese long’ inbred line 9930, version 2 [30]) using Bowtie, version 2.2.6 [56]. Filtering the reads for the genomic location of *CsaMLO1* and *CsaMLO11* and SNP/indel calling were performed using the SAMtools software package, version 0.1.18 [57].

To inspect a region with low read coverage in intron 6 of *CsaMLO1,* this region was amplified by PCR from DNA of genotype CS-PMR1 using primers 5′-CCTGCCTTGATGTGGATCGT-3′ (Forward) and 5′-AGTGCCTTCTTCTGACCGTT-3′ (Reverse). To inspect a region with low read coverage in intron 12 of *CsaMLO11,* this region was amplified by PCR from DNA of genotype CS-PMR1 using primers 5′- AGCACACAGAGGATTTGGTCA-3′ (Forward) and 5′-TGAACGAGAACCCTGATGCA-3′ (Reverse). For both PCR reactions, as template 2 μl DNA was used in a 50 μl reaction using DreamTaq DNA polymerase (ThermoFisher Scientific, U.S.A.). Cycling conditions were: 1 min initial denaturation at 95 °C, followed by 40 cycles of 30 s denaturation at 95 °C, 30 s annealing at 60 °C and 1 min annealing at 72 °C. Reactions were finished by 7 min incubation at 72 °C. PCR products were visualised by staining with GelRed and electrophoresis on agarose gels. Sequencing reactions were performed in triplicate, using the same primers used for amplification (GATC Biotech, Germany). Obtained sequences were aligned using CLC Main Workbench v. 7.6.4. The consensus sequence for the amplified region was extracted from the alignment. This consensus sequences were then aligned to the genomic reference sequences of *CsaMLO1* and *CsaMLO11*, respectively.

To verify whether the observed sequence variants in intron 6 of *CsaMLO1* and intron 12 of *CsaMLO11* in genotype CS-PMR1 cause any effect on splicing, the corresponding regions were amplified from cDNA. RNA was isolated from young leaves of cucumber genotypes CS-PMR1 and Santou using the RNeasy Kit (Qiagen, Germany). Possible DNA contamination of RNA samples was removed by treatment with DNase I, Amp. Grade (Invitrogen life technologies, U.S.A.). cDNA was synthesised using 2 μg of RNA samples with an iScript cDNA Synthesis Kit (Bio-Rad Laboratories, U.S.A.). A *CsaMLO1* coding sequence fragment was amplified from cDNA using primers *CsaMLO1-*Forward (as above) and 5′-ATGGCAGCCATAGATACGCC (Reverse). A *CsaMLO11* coding sequence fragment was amplified from cDNA using primers 5′-GTGGTGGTCAGTATCAGCCC-3′ (Forward) and 5′-CGTCGAACCCATCTGTGTGA-3′ (Reverse). PCR reactions were performed using DreamTaq DNA polymerase (ThermoFisher Scientific, U.S.A.), with cycling conditions as described above. PCR products were visualised by staining with GelRed and electrophoresis on agarose gels. Sequencing reactions were performed in triplicate, using the same primers used for amplification (GATC Biotech, Germany). Obtained sequences were aligned using CLC Main Workbench v. 7.6.4. The consensus sequence for the amplified region was extracted from the alignment. These consensus sequences were then aligned to the cDNA reference sequences of *CsaMLO1* and *CsaMLO11*, respectively.

## Additional files

Additional file 1: The relative transcript abundances of *CsaMLO1*, *CsaMLO8* and *CsaMLO11* in T2 families of a tomato *mlo* mutant overexpressing *CsaMLO1*, *CsaMLO8* and *CsaMLO11,* were determined by qRT-PCR. Data were normalised relatively to the reference gene *SlEF-α*. Average transcript abundances of four or five randomly selected individuals are shown on a logarithmic scale. Error bars represent standard error of the mean. Different letters above the bars indicate statistical significance of Ct-values (One-way ANOVA with Dunnet’s T3 post hoc test, *P* < 0.05). *CsaMLO1*, *CsaMLO8* or *CsaMLO11* expression was not detectable in non-transformed tomato *mlo* mutant or Moneymaker. (PDF 341 kb)

Additional file 2: Data on the transcript abundance in four tissues of *Arabidopsis thaliana*, determined using RNA-seq was investigated and downloaded using the Expression Atlas of EMBL-EBI (https://www.ebi.ac.uk/gxa/home). The FPKM values (Fragments Per Kilobase of transcript per Million mapped fragments) for *AtMLO2*, *AtMLO6* and *AtMLO12* in each of the tissues is shown on a logarithmic scale. (PDF 350 kb)

Additional file 3: Relative transcript abundances in two tissues of PM susceptible cucumber cultivar ‘Sheila’ (A) hypocotyl and B) leaf, before and at 4, 6, 8, 12 and 24 h post inoculation with *P. xanthii* were determined using qRT-PCR. Data were normalized relative to the geometric average of the Ct values of reference genes *Ef-α* and *CACS*, and subsequently normalized relative to the average dCt value at 0 hpi for both tissues. Each bar shows the relative expression of five to eight biological replicates, as indicated above the bars, on a logarithmic scale. Error bars indicate standard error of the mean. (PDF 374 kb)

Additional file 4: The list of SNPs and indels in 115 resequenced cucumber accessions, available from the Cucurbit Genomics Database [38], was filtered for the genomic region of *CsaMLO1* (Chr1:8,159,428.8165253). The genic location of each SNP/Indel was determined manually (exon/intron), for exonic SNPs the effect on the predicted amino acid sequence was scored (synonymous/nonsynonymous). (XLSX 57 kb)

Additional file 5: The list of SNPs and indels in 115 resequenced cucumber accessions, available from the Cucurbit Genomics Database [38], was filtered for the genomic region of *CsaMLO11* (Chr6:14,120,024.14,125,039). The genic location of each SNP/Indel was determined manually (exon/intron), for exonic SNPs the effect on the predicted amino acid sequence was scored (synonymous/nonsynonymous). (XLSX 50 kb)

Additional file 6: Protein alignment of clade V MLO proteins of *Arabidopsis thaliana* (AtMLO2, 6 and 12), *Medicago trunculata* (MtMLO1), *Pisum sativum* (PsMLO1), *Lotus japonicus* (LjMLO1), *Cucumis sativus* (CsaMLO1, 8 and 11), *Solanum lycopersicum* (SlMLO1), *Capsicum annuum* (CaMLO2) and *Nicotiana tabacum* (NtMLO1). A bar graph shows the conservation of the individual residues. Colours indicate amino acid residues with similar physiochemical properties according to the RasMol colour scheme. Locations of amino acid substitutions in CsaMLO1 due to SNPs are indicated by a red arrow. (PDF 3224 kb)

Additional file 7: Resequencing data of the genomic region of *CsaMLO1* in cucumber genotypes CS-PMR1 and Santou. The location of the gene on the chromosome is indicated by a red cursor. For each of the two genotypes, the total reads mapping to the location and the coverage per base pair are given. SNPs are indicated by coloured stripes (green for A, red for T, blue for C, brown for G), indels are indicated by black stripes. Read pairs with a small (smallest 0.5%) or large (largest 0.5%) insert size are coloured dark blue or dark red, respectively. Reads for which the other mate in the mate pair was not mapped are indicated by a bright red outline. (PDF 356 kb)

Additional file 8: Resequencing data of the genomic region of *CsaMLO11* in cucumber genotypes CS-PMR1 and Santou. The location of the gene on the chromosome is indicated by a red cursor. For each of the two genotypes, the total reads mapping to the location and the coverage per base pair are given. SNPs are indicated by coloured stripes (green for A, red for T, blue for C, brown for G), indels are indicated by black stripes. Read pairs with a small (smallest 0.5%) or large (largest 0.5%) insert size are coloured dark blue or dark red, respectively. Reads for which the other mate in the mate pair was not mapped are indicated by a bright red outline. (PDF 356 kb)

Additional file 9: The list of SNPs and indels identified in the genomic sequence of *CsaMLO1* and *CsaMLO11* in the resequenced cucumber genotypes CS-PMR1 and Santou. The reference allele (in cucumber reference genome Chinese Long 9930) and the alternative allele are given. Furthermore, the genic location of each SNP/Indel was determined (exon/intron), for exonic SNPs the effect on the predicted amino acid sequence was scored (synonymous/nonsynonymous). (XLSX 12 kb)

Additional file 10: Intron 6 of *CsaMLO1* (A) and intron 12 of *CsaMLO11* (D) were amplified from genomic DNA isolated from the cucumber genotype CS-PMR1, and subsequently sequenced in triplicate by Sanger sequencing. The obtained sequences were aligned to the reference cucumber genome (Chinese long inbred 9930, v2). Numbers above the alignment are relative to the start codon of the respective genes. The region surrounding intron 6 of *CsaMLO1* (B and C) and the region surrounding intron 12 of *CsaMLO11* (E and F) were amplified from cDNA of cucumber genotypes CS-PMR1 and Santou, and subsequently sequenced in triplicate by Sanger sequencing. Amplified products were analysed on 1.25% agarose gels. It was found that for both amplified regions, the products amplified from CS-PMR1 and Santou were of similar sizes. Sequences of cDNA were identical to one another and to the reference cDNA sequence (Chinese long inbred 9930, v2). (PDF 2940 kb)

Additional file 11: Relative transcript abundances of *CsaMLO1* and *CsaMLO11* in leaf tissues of cucumber genotypes Sheila, Santou and CS-PMR1 were determined using qRT-PCR. Data were normalized relative to the geometric average of the Ct values of reference genes *Ef-α*, *TIP41* and *CACS*, and subsequently normalized relative to the average dCt value of Sheila. Each bar shows the relative expression of three biological replicates, on a logarithmic scale. Error bars indicate standard error of the mean. (PDF 349 kb)
