# Supplementary figures and images for: Functional characterization of cucumber (Cucumis sativus L.) Clade V MLO genes
Source: BMC Plant Biol. 2017 Apr 21;17:80. doi: 10.1186/s12870-017-1029-z (PMC5399834; doi:10.1186/s12870-017-1029-z)

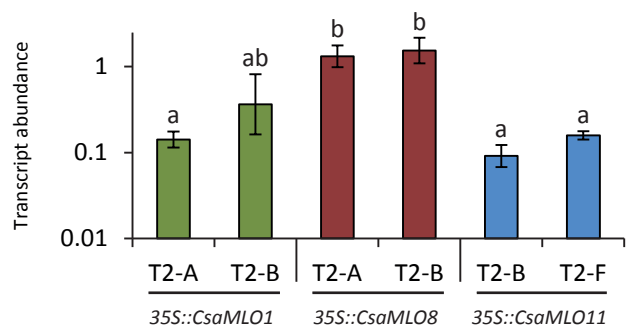

Supplement: Supplementary file 1 — The relative transcript abundances of CsaMLO1, CsaMLO8 and CsaMLO11 in T2 families of a tomato mlo mutant overexpressing CsaMLO1, CsaMLO8 and CsaMLO11, were determined by qRT-PCR. Data were normalised relatively to the reference gene SlEF-α. Average transcript abundances of four or five randomly selected individuals are shown on a logarithmic scale. Error bars represent standard error of the mean. Different letters above the bars indicate statistical significance of Ct-values (One-way ANOVA with Dunnet’s T3 post hoc test, P < 0.05). CsaMLO1, CsaMLO8 or CsaMLO11 expression was not detectable in non-transformed tomato mlo mutant or Moneymaker. (PDF 341 kb) [file 12870_2017_1029_MOESM1_ESM.pdf]

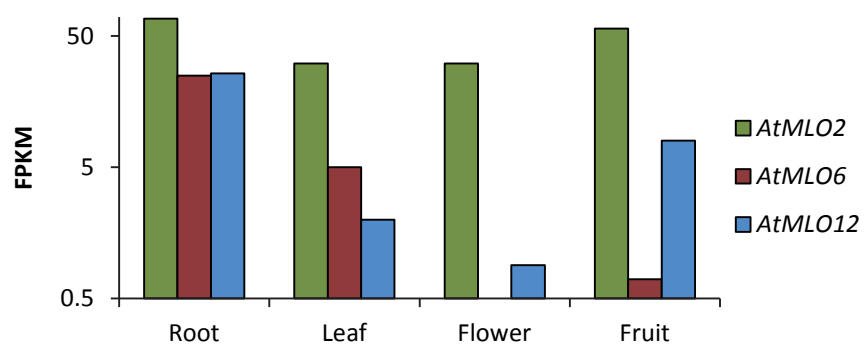

Supplement: Supplementary file 2 — Data on the transcript abundance in four tissues of Arabidopsis thaliana, determined using RNA-seq was investigated and downloaded using the Expression Atlas of EMBL-EBI (https://www.ebi.ac.uk/gxa/home). The FPKM values (Fragments Per Kilobase of transcript per Million mapped fragments) for AtMLO2, AtMLO6 and AtMLO12 in each of the tissues is shown on a logarithmic scale. (PDF 350 kb) [file 12870_2017_1029_MOESM2_ESM.pdf]

**A**

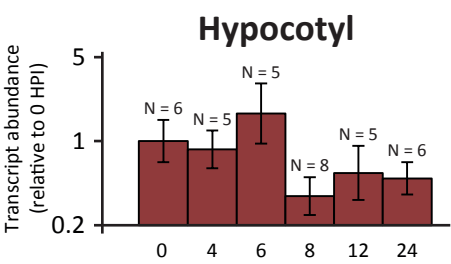

**B**

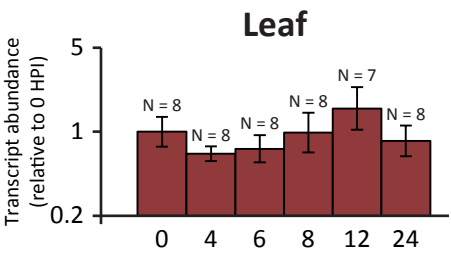

Supplement: Supplementary file 3 — Relative transcript abundances in two tissues of PM susceptible cucumber cultivar ‘Sheila’ (A) hypocotyl and B) leaf, before and at 4, 6, 8, 12 and 24 h post inoculation with P. xanthii were determined using qRT-PCR. Data were normalized relative to the geometric average of the Ct values of reference genes Ef-α and CACS, and subsequently normalized relative to the average dCt value at 0 hpi for both tissues. Each bar shows the relative expression of five to eight biological replicates, as indicated above the bars, on a logarithmic scale. Error bars indicate standard error of the mean. (PDF 374 kb) [file 12870_2017_1029_MOESM3_ESM.pdf]

*CsaMLO1*

CS-PMR1

Santou

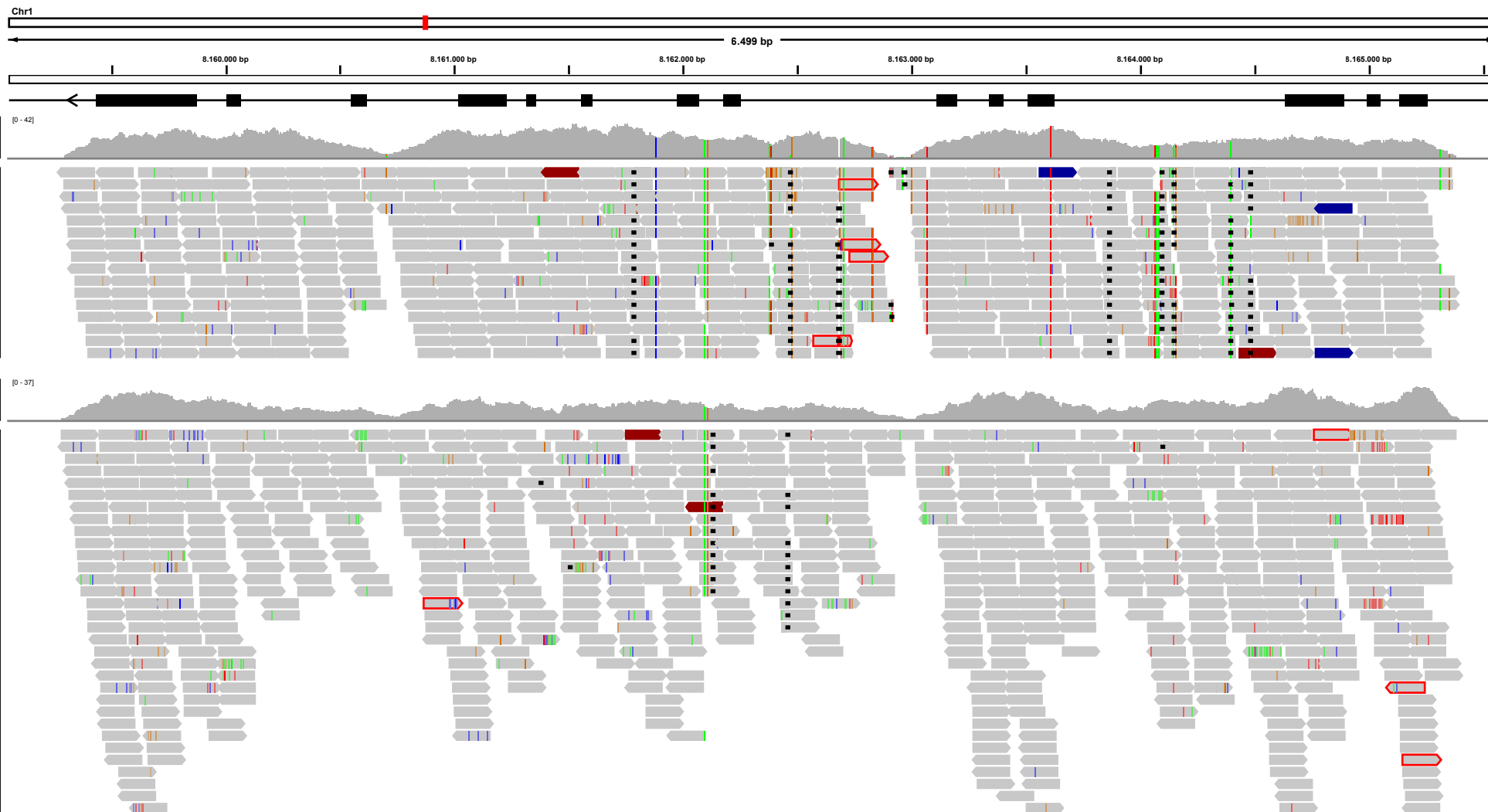

Supplement: Supplementary file 7 — Resequencing data of the genomic region of CsaMLO1 in cucumber genotypes CS-PMR1 and Santou. The location of the gene on the chromosome is indicated by a red cursor. For each of the two genotypes, the total reads mapping to the location and the coverage per base pair are given. SNPs are indicated by coloured stripes (green for A, red for T, blue for C, brown for G), indels are indicated by black stripes. Read pairs with a small (smallest 0.5%) or large (largest 0.5%) insert size are coloured dark blue or dark red, respectively. Reads for which the other mate in the mate pair was not mapped are indicated by a bright red outline. (PDF 356 kb) [file 12870_2017_1029_MOESM7_ESM.pdf]

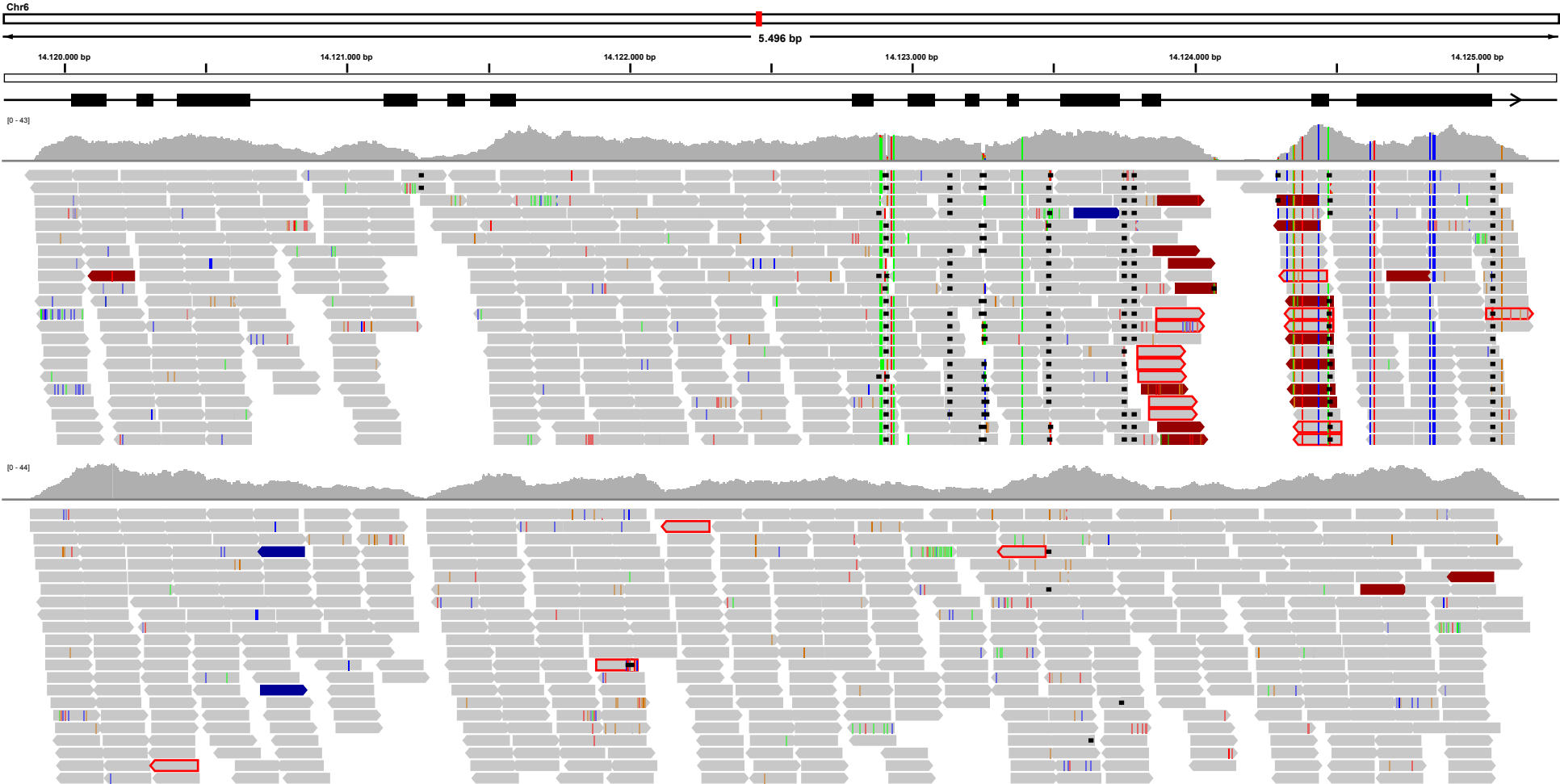

Supplement: Supplementary file 8 — Resequencing data of the genomic region of CsaMLO11 in cucumber genotypes CS-PMR1 and Santou. The location of the gene on the chromosome is indicated by a red cursor. For each of the two genotypes, the total reads mapping to the location and the coverage per base pair are given. SNPs are indicated by coloured stripes (green for A, red for T, blue for C, brown for G), indels are indicated by black stripes. Read pairs with a small (smallest 0.5%) or large (largest 0.5%) insert size are coloured dark blue or dark red, respectively. Reads for which the other mate in the mate pair was not mapped are indicated by a bright red outline. (PDF 356 kb) [file 12870_2017_1029_MOESM8_ESM.pdf]

**A**

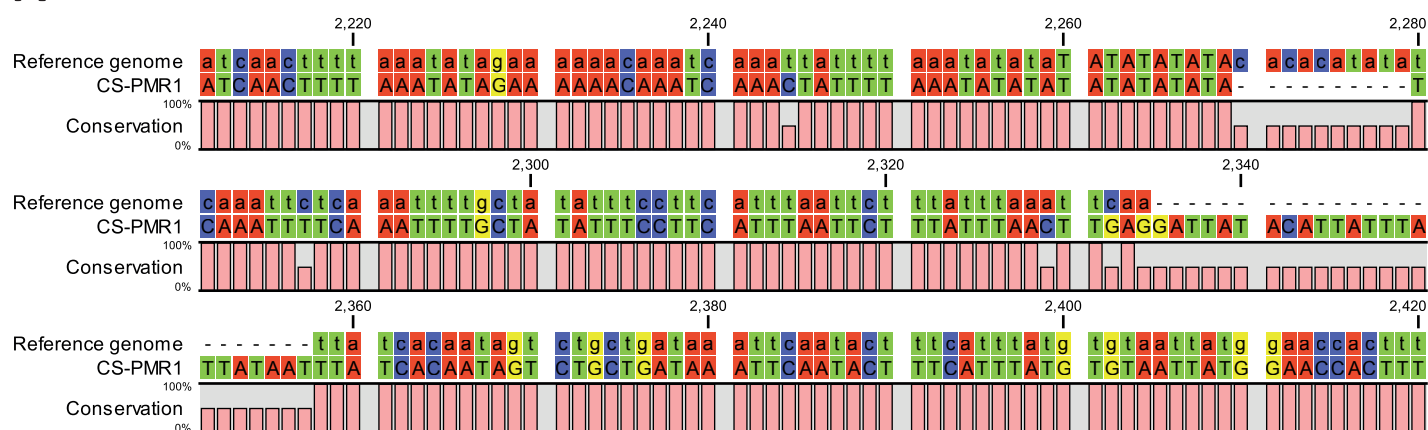

**B**

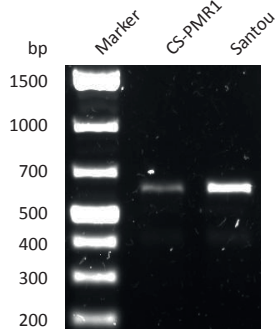

**C**

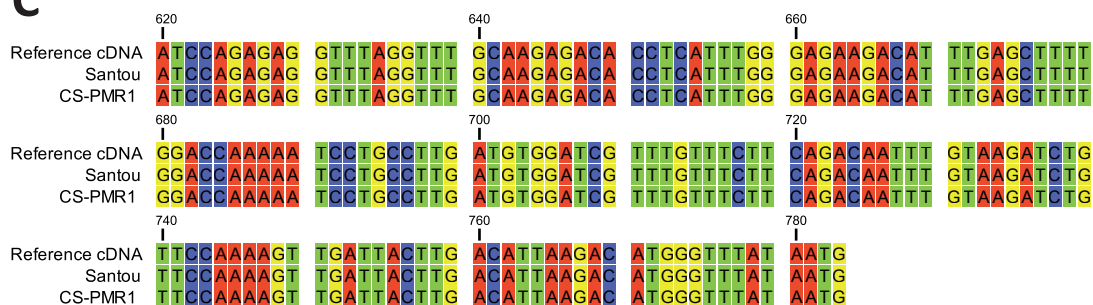

**D**

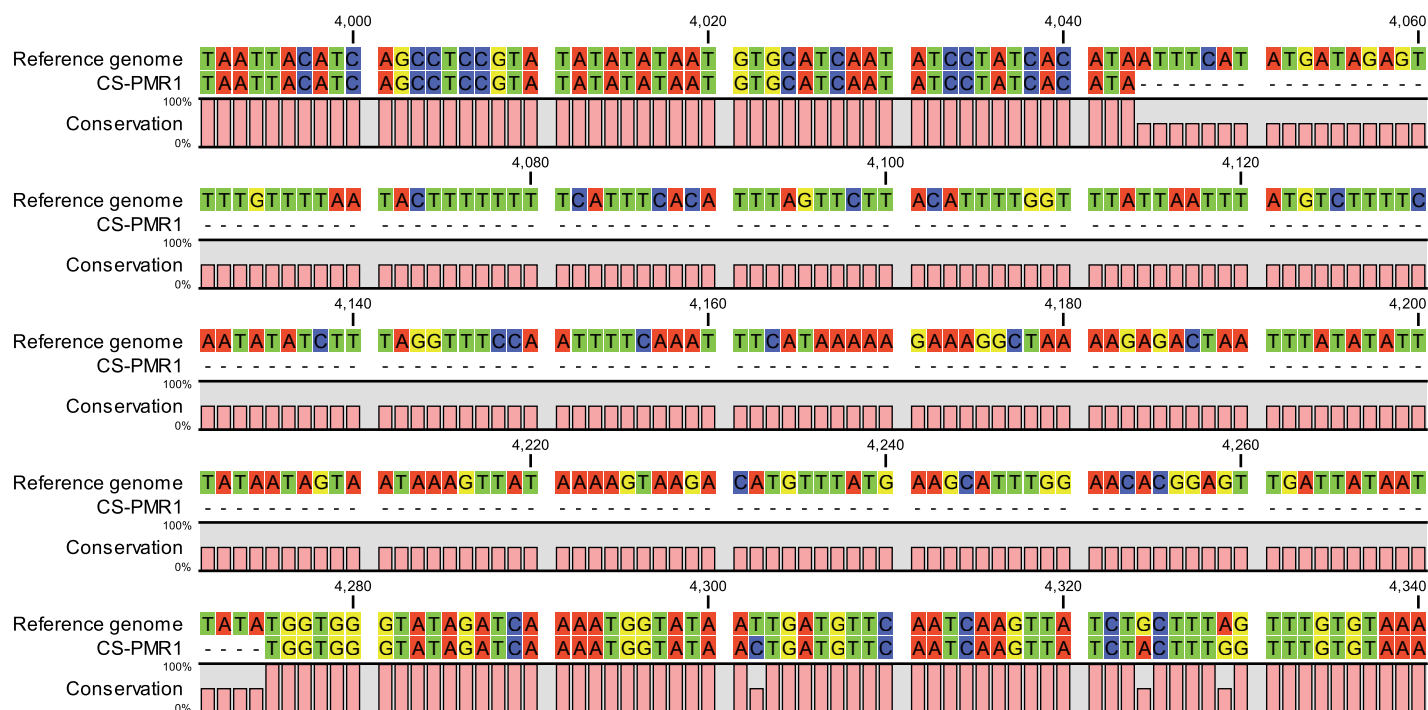

**E**

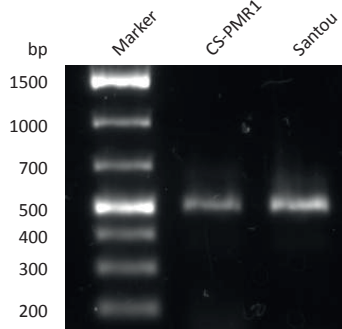

**F**

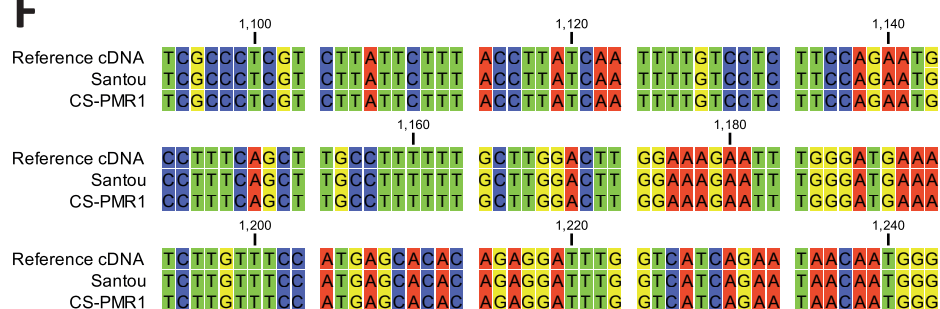

Supplement: Supplementary file 10 — Intron 6 of CsaMLO1 (A) and intron 12 of CsaMLO11 (D) were amplified from genomic DNA isolated from the cucumber genotype CS-PMR1, and subsequently sequenced in triplicate by Sanger sequencing. The obtained sequences were aligned to the reference cucumber genome (Chinese long inbred 9930, v2). Numbers above the alignment are relative to the start codon of the respective genes. The region surrounding intron 6 of CsaMLO1 (B and C) and the region surrounding intron 12 of CsaMLO11 (E and F) were amplified from cDNA of cucumber genotypes CS-PMR1 and Santou, and subsequently sequenced in triplicate by Sanger sequencing. Amplified products were analysed on 1.25% agarose gels. It was found that for both amplified regions, the products amplified from CS-PMR1 and Santou were of similar sizes. Sequences of cDNA were identical to one another and to the reference cDNA sequence (Chinese long inbred 9930, v2). (PDF 2940 kb) [file 12870_2017_1029_MOESM10_ESM.pdf]

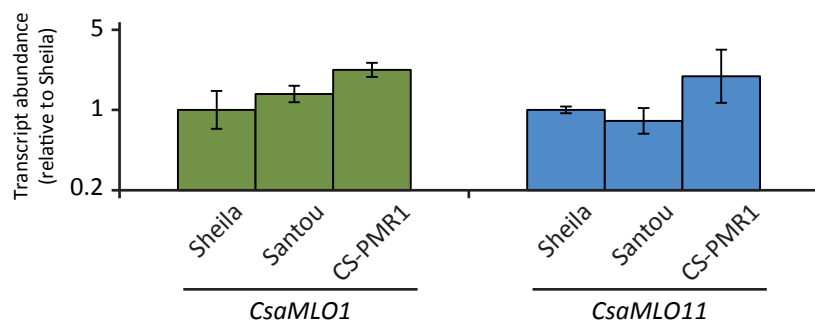

Supplement: Supplementary file 11 — Relative transcript abundances of CsaMLO1 and CsaMLO11 in leaf tissues of cucumber genotypes Sheila, Santou and CS-PMR1 were determined using qRT-PCR. Data were normalized relative to the geometric average of the Ct values of reference genes Ef-α, TIP41 and CACS, and subsequently normalized relative to the average dCt value of Sheila. Each bar shows the relative expression of three biological replicates, on a logarithmic scale. Error bars indicate standard error of the mean. (PDF 349 kb) [file 12870_2017_1029_MOESM11_ESM.pdf]
