# Supplementary material for: Functional characterization of cucumber (Cucumis sativus L.) Clade V MLO genes
Source: BMC Plant Biol. 2017 Apr 21;17:80. doi: 10.1186/s12870-017-1029-z (PMC5399834; doi:10.1186/s12870-017-1029-z)
Supplement: Supplementary file 6 — Protein alignment of clade V MLO proteins of Arabidopsis thaliana (AtMLO2, 6 and 12), Medicago trunculata (MtMLO1), Pisum sativum (PsMLO1), Lotus japonicus (LjMLO1), Cucumis sativus (CsaMLO1, 8 and 11), Solanum lycopersicum (SlMLO1), Capsicum annuum (CaMLO2) and Nicotiana tabacum (NtMLO1). A bar graph shows the conservation of the individual residues. Colours indicate amino acid residues with similar physiochemical properties according to the RasMol colour scheme. Locations of amino acid substitutions in CsaMLO1 due to SNPs are indicated by a red arrow. (PDF 3224 kb) [file 12870_2017_1029_MOESM6_ESM.pdf]

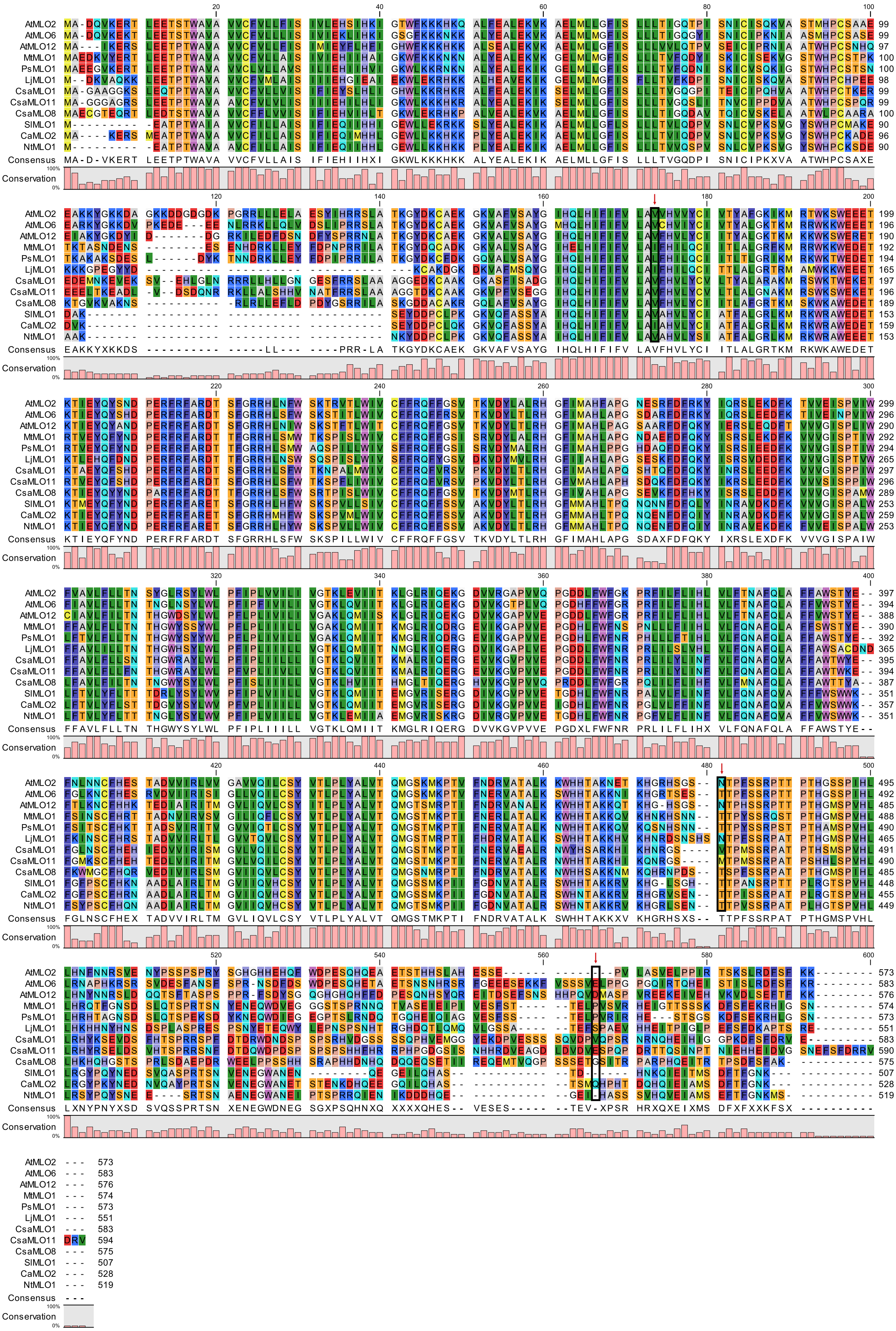
AtMLO2
AtMLO6
AtMLO12
MtMLO1
PsMLO1
LjMLO1
CsaMLO1
CsaMLO11
CsaMLO8
SlMLO1
CaMLO2
NtMLO1
Consensus
Conservation
